# *Burkholderia cenocepacia* Vaginal Infection in Patient with Smoldering Myeloma and Chronic Hepatitis C

**DOI:** 10.3201/eid1011.040127

**Published:** 2004-11

**Authors:** Andrea Petrucca, Paola Cipriani, Rosa Sessa, Antonella Teggi, Rosalia Pustorino, Daniela Santapaola, Mauro Nicoletti

**Affiliations:** * II Facoltà di Medicina e Chirurgia dell'Università "La Sapienza," Rome, Italy;; † I Facoltà di Medicina e Chirurgia dell'Università "La Sapienza," Rome, Italy;; ‡ Università "G. D'Annunzio," Chieti, Italy

**Keywords:** Burkholderia cenocepacia, smoldering myeloma, SMM, hepatitis C virus, HCV, dispatch

## Abstract

We report a case of a vaginal infection caused by a strain of *Burkholderia cenocepacia*. The strain was isolated from vaginal swab specimens from a 68-year-old woman with smoldering myeloma and chronic hepatitis C virus infection who was hospitalized for abdominal abscess. Treatment with piperacillin/tazobactam eliminated *B. cenocepacia* infection and vaginal symptoms.

Members of genus *Burkholderia* are aerobic, non–spore-forming, catalase-positive, gram-negative bacteria; most are oxidase positive (*1*). This genus comprises opportunistic pathogens responsible for important infections in immunocompromised persons and in cystic fibrosis (CF) patients (*2**,**3*). To date, the genus *Burkholderia* comprises more than 30 species, including the *Burkholderia cepacia* complex, *B. mallei*, and *B. pseudomallei* (*2*). The *B. cepacia* complex is a group of microorganisms composed of at least nine closely related genomovars (*2**,**3*). All genomovars have been shown to cause infections, and *B. cenocepacia* and *B. multivorans* (genomovars III and II, respectively) are the genomovars most frequently isolated from CF patients (*4**–**7*).

Nosocomial infections caused by *B. cepacia* complex have been reported in non-CF patients, principally associated with the use of contaminated disinfectants, anaesthetic solutions, and invasive treatments such as urinary and intravenous catheterization (*8*). These strains are intrinsically resistant to most antimicrobial agents and are difficult to eliminate (*8**,**9*). Cases of *B. cepacia* complex infections are underestimated because of the complex taxonomy of this genus and the poor sensitivity and specificity of commercial identification systems (*10*). Recently, molecular methods, mainly polymerase chain reaction (PCR)-based, have been developed to circumvent this issue (*10**–**13*).

We report a case of vaginal infection, caused by *B. cenocepacia*, in a patient affected by smoldering myeloma, and chronic hepatitis C virus (HCV) infection. Bacterial identification at species level was assessed by four combined PCR–based molecular methods. Therapy based on treatment with piperacillin/tazobactam completely eliminated the infection as well as the vaginal symptoms.

## Case Report

In August 2003, a 68-year-old woman with smoldering myeloma and chronic HCV infection (the patient had cirrhosis since 1994) was admitted to the "Sant'Andrea" Hospital (2nd Faculty of Medicine, "La Sapienza" University, Rome, Italy), with a 15-day history of fever, malaise, asthenia, fatigue, abdominal pain, and swelling of from lower limbs. One week before admission, she had been treated with ciprofloxacin (500 mg twice a day) without improvement of any of the clinical symptoms. On admission (day 1), the patient had a fever (38.4°C) and showed abundant ascitic fluid and jaundice. Laboratory values were indicative of macrocytic anemia (erythrocytes, 3.6 x 10^9^/L; hemoglobin, 110 g/L; mean corpuscular volume, 103 fL; hematocrit, 31%; serum iron level, 10.74 µmol/L; and serum ferritin level, 170 µg/L. Platelet count was 46 x 10^9^/L, and leukocyte count was 14 x 10^9^/L with neutrophils (13 x 10^9^/L) and lymphocytes (0.5 x 10^9^/L). The patient had high values of the erythrocyte sedimentation rate (ESR) in the first hour (71 mm/h) and C-reactive protein (CRP) (4.1 mg/L). Increased total serum proteins (8.1 g/dL) and hypoalbuminemia (23 g/L) were also detected. Six blood samples were taken at 3-hour intervals during the first day of hospitalization and cultured to detect the growth of aerobic and anaerobic microorganisms (Bactec System, Becton Dickinson, Sparks, MD). All blood cultures were negative. Abdominal ecographic and tomographic scans showed a pseudocystic formation in the pancreas. The pancreatic formation was drained because surgical intervention was not appropriate for the patient. On admission day 2, the patient was transferred to the Infectious Diseases Unit; there, intensive strong diuretic therapy was initiated, and a urinary catheter was inserted. Results of microbiologic analysis of urine and of a liquid taken from the pseudocystic formation were negative for common pathogenic bacteria. In spite of these results, the patient was given intravenous amoxicillin/clavulananate (1.2 g three times a day) and amikacin (1 g once a day) (day 3). After 5 days of antimicrobial drug therapy, the clinical symptoms of the patient slightly improved. On day 9 (i.e., after 6 days of antimicrobial drug therapy and 7 days of urinary catheterization), the patient exhibited an abundant white vaginal discharge with vulvar pain and burning. Vaginal swabs were streaked on different selective media. Columbia agar base, supplemented with 5% (vol/vol) sheep blood, and MacConkey agar plates showed a monomicrobic culture constituted by catalase-positive and oxidase-positive gram-negative rods that did not grow under anaerobic conditions. A presumptive identification of *B. cepacia* was made by using the API20NE (bioMérieux, Marcy l'Etoile, France), while the Vitek 2.0 identification system (bioMérieux) did not recognize the isolate as *B. cepacia* (*10*). Identification at the species level was achieved by four different PCR-based combined molecular methods, namely, *Dde*I and *Hae*III restriction fragment length polymorphisms (RFLP) of 16S rDNA and *recA* gene analysis, *recA* genomovar-specific PCR, and *recA* sequence analysis (*11**–**13*). Control strains belonging to different genomovars of *B. cepacia* complex were included in all molecular analyses (*14*). Bacterial genomic DNA was extracted by using a commercial kit (Qiagen genomic-tip, Qiagen Inc., Hilden, Germany) as previously described (*14*). The bacterial isolate showed a 16S rRNA *Dde*I-RFLP pattern 1 and a *recA Hae*III-RFLP pattern H (data not shown; 14), patterns indicative of *B. cenocepacia* (*11**–**14*). Genomovar-specific PCR was performed with primer pairs annealing to internal regions of the *recA* gene (*12**,**13*). A DNA fragment with a molecular mass of approximately 800 bp, consistent with the expected 781-bp *B. cenocepacia* amplification fragment, was successfully amplified with the primer pair BCRG3B1/BCRG3B2 (data not shown) (*12**,**13*). To unambiguously identify the bacterial isolate, we sequenced the amplified *recA* DNA fragment that was subjected to *recA Hae*III-RFLP analysis (*13*). The *recA* DNA sequence of the bacterial isolate (GenBank accession no. AJ786367), subjected to BLAST analysis (http://www.ncbi.nlm.nih.gov/BLAST), showed >99% homology with the *recA* sequence of the *B. cenocepacia* reference strain LMG 18829 (GenBank accession no. AF143784). Phylogenetic analysis, based on *recA* DNA sequences, indicated that the clinical isolate belonged to the *B. cenocepacia* (genomovar III, lineage IIIB) (Figure) (*4**,**13*).

**Figure:**
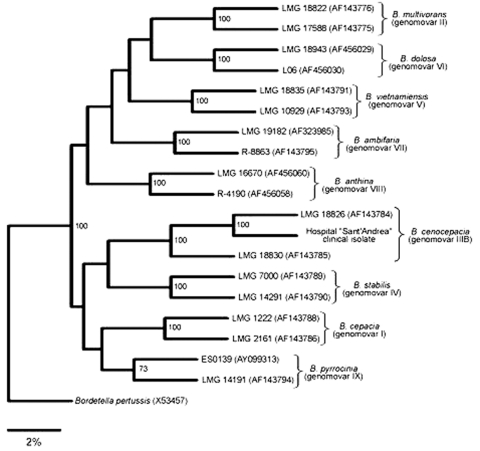
The consensus phylogenetic tree of *recA* DNA sequences of *Burkholderia cepacia* complex strains, representative of each genomovar, and of the *B. cenocepacia* isolate (GenBank accession no. AJ786367) was constructed with the PHYLIP package (version 3.6) (http://evolution.genetics.washington.edu/phylip.html). Only *recA* DNA sequences of reference *B. cenocepacia* strains (genomovar III, lineage IIIB) are included in the tree (*4**,**13*). Alignments were performed with the Clustal W program. Genetic distance is indicated on the scale, and bootstrap analysis for nodes values >70% are shown. GenBank accession numbers of the *recA* DNA sequences are shown for each reference strains.

The *B. cenocepacia* isolate was resistant to penicillin, mezlocillin, piperacillin, amoxicillin/clavulanate, nitrofurantoin, ciprofloxacin, carbapenems, cephalosporins, aminoglycosides, and tetracycline and sensitive to trimethoprim/sulfamethoxazole and piperacillin/tazobactam. When the antimicrobial drug susceptibility profile was considered, the amoxicillin/clavulanate and amikacin antibiotic therapy was interrupted, and intravenous piperacillin/tazobactam combination was administered (4.5 g, three times a day for 4 weeks). Vaginal swabs were taken every 3 days during the 4 weeks of the antimicrobial drug therapy and, afterwards, every 20 days for a total follow-up period of 3 months. After 10 days of the piperacillin/tazobactam treatment, vaginal symptoms disappeared and cultured vaginal swabs did not show *B. cenocepacia*. After the piperacillin/tazobactam treatment ended, the patient did not exhibit any signs of vaginal infection.

## Conclusions

We think this is the first description of a vaginal infection caused by *B. cenocepacia*. The patient's immunodepression from smoldering myeloma and chronic HCV likely favored vaginal colonization by *B. cenocepacia*. Urinary catheterization might have favored vaginal colonization by *B. cenocepacia*, even if we did not isolate *B. cenocepacia* from catheters, disinfectants, and selected hospital environmental samples analyzed from October 2003 to February 2004 (*15*). Moreover, the antimicrobial agents, ciprofloxacin, and amoxicillin/clavulanate and amikacin, administered to the patient before and during hospitalization, might also have altered the patient's vaginal flora. Piperacillin/tazobactam eliminated vaginal symptoms and *B. cenocepacia* from the vaginal mucosa, thus indicating that the detected isolate was indeed responsible for the infection.

Microorganisms belonging to the *B. cepacia* complex are difficult to identify by conventional biochemical tests and commercial systems (*8*). This case report highlights the importance of the use of molecular techniques to quickly and accurately identify members of the *B. cepacia* complex (*10**,**13*). The ability of *B. cenocepacia* to cause vaginal infections is unusual. Further studies are needed to clarify whether specific virulence factors are carried and expressed by the *B. cenocepacia* clinical isolate, conferring to this strain the specific ability to colonize and multiply within the vaginal mucosa.
